# Health, function and disability in stroke patients in the community

**DOI:** 10.1590/bjpt-rbf.2014.0171

**Published:** 2016-06-20

**Authors:** Bárbara P. B. Carvalho-Pinto, Christina D. C. M. Faria

**Affiliations:** 1 Escola de Educação Física, Fisioterapia e Terapia OcupacionalUniversidade Federal de Minas GeraisUniversidade Federal de Minas GeraisBelo HorizonteMGBrazilDepartamento de Fisioterapia, Escola de Educação Física, Fisioterapia e Terapia Ocupacional, Universidade Federal de Minas Gerais (UFMG), Belo Horizonte, MG, Brazil

**Keywords:** rehabilitation, stroke, international classification of functioning, disability and health, continuity of patient care

## Abstract

**Background:**

Stroke patients commonly have impairments associated with reduction in functionality. Among these impairments, the motor impairments are the most prevalent. The functional profile of these patients living in the community who are users of the primary health-care services in Brazil has not yet been established

**Objective:**

To describe the functional profile of stroke patients who are users of the primary health-care services in Brazil, looking at one health-care unit in the city of Belo Horizonte, Brazil.

**Method:**

From medical records and home visits, data were collected regarding health status, assistance received following the stroke, personal and environmental contextual factors, function and disability, organized according to the conceptual framework of the International Classification of Functioning, Disability and Health (ICF). Test and instruments commonly applied in the assessment of stroke patients were used.

**Results:**

Demographic data from all stroke patients who were users of the health-care unit (n=44, age: 69.23±13.12 years and 67±66.52 months since the stroke) participated of this study. Most subjects presented with disabilities, as changes in emotional function, muscle strength, and mobility, risks of falling during functional activities, negative self-perception of quality of life, and perception of the environment factors were perceived as obstacles. The majority of the patients used the health-care unit to renew drug prescriptions, and did not receive any information on stroke from health professionals, even though patients believed it was important for patients to receive information and to provide clarifications.

**Conclusion:**

Stroke patients who used primary health-care services in Brazil have chronic disabilities and health needs that require continuous health attention from rehabilitation professionals. All of these health needs should be considered by health professionals to provide better management as part of the integral care of stroke patients, as recommended by the clinical practice guidelines for stroke rehabilitation.

## BULLET POINTS

- Stroke patients who use the primary health-care services in Brazil showed several disabilities.
- Impairments in emotional and motor functions were common in these patients.
- Changes in mobility and risks of falling were common in these patients.
- Environmental factors were perceived as obstacle by these patients.
- Primary health-care services were primarily used by these patients to renew drug prescriptions.

## Introduction

Stroke has a very high incidence and prevalence, and is one the main causes of disability worldwide^1^^,^^2^. Approximately 90% of stroke survivors have compromised functions^3^^,^^4^. To reduce this impact, it is necessary for health-care professionals, including physical therapists, to provide adequate follow-up and to assistance to patients in recovering their health and functionality, to prevent further diseases and disability, and to promote health and functionality^3^^,^^5^.

Clinical practice guidelines for stroke rehabilitation recommend the following: systematic follow-up of patients by a multiprofessional team, provision of rehabilitation services^5^^,^^6^, participation of patients in continued physical exercise programs offered in the community to maintain their function and health, and assessment of patients by rehabilitation professionals at least once a year to verify the need for new interventions^6^. To better direct the treatment approach and clinical decision-making strategies, the rehabilitation procedures recommended by these guidelines^5^^,^^6^ should be guided by the Model of Functioning and Disability related to the International Classification of Functioning, Disability, and Health (ICF)^7^. The ICF provides guidelines for the identification of the functional profile of each patient, allowing for the application of the proper approaches and clinical decision-making strategies^8^_._

Identification of the health and function profile of the population of a certain region may enable identifying the common needs of these patients. This allows for a better orientation of the care offered to people living in the same area who have similar conditions, and the identification of possible improvement or modification in the treatment strategies for this population^9^. For this purpose, studies that show results beyond the features of persons in a convenience sample are necessary. In addition to assessing persons with common health conditions (e.g., stroke), it is also necessary to investigate their access to health-care services.

In this context, there has been great interest in information related to the function and health profile of Brazilian stroke patients. Although previous studies have aimed to describe this profile, they either included convenience samples^10^^,^^11^, which limited the findings and conclusions, or chose a theoretical model (to describe the profile) that did not include biopsychosocial variables^12^^-^^14^. Thus, in this study, the function, disability, and health profile of stroke patients will be described using the Primary Health-Care of the Brazilian Unified Health System (SUS), with a health-care unit located in the city of Belo Horizonte, Brazil as a reference.

## Method

A descriptive study was used in which all stroke patients who used the selected health-care unit were invited to participate. The health-care unit was selected on the basis of the criteria established by the Brazilian Ministry of Health (BMH), as follows: a multi professional team responsible for a maximum of 4,000 persons in a defined area; composed of a doctor, a nurse, a nursing assistant, and health-care community agents (HCAs); and with sufficient HCAs to cover 100% of the registered population^15^^,^^16^, in addition to the support of a team from the Family Health-Care Support Centre, organized in compliance with the recommendations of the BMH^16^. Finally, a health-care unit located in the northeast sanitary district of the city of Belo Horizonte, MG, Brazil was selected.

At the time of data collection, the selected health-care unit assisted a total population of 16,363 users including stroke patients, and had four family health-care teams, in addition to a team from the Family Health-Care Support Centre, comprising a social worker, a physical educator, two physical therapists, one speech therapist, one nutritionist, one psychologist, one psychiatrist, and one occupational therapist. One of the physical therapists in the Family Health-Care Support Centre worked exclusively at the health-care unit.

### Sample

At least once a month, for 1 year, one of the members of the research group participated in the meetings of the family health-care teams to identify potential participants in the study, using the the following inclusion criteria: suffered a primary stroke or a clinical diagnosis of recurring stroke for >6 months; lived in the community serviced by the health-care unit; used the SUS while being registered at the health-care unit, as verified by an employee of the health-care unit; age ≥20 years; and had freely signed the clarified consent term approved by the research ethics committee of the Universidade Federal de Minas Gerais (UFMG), Belo Horizonte, MG, Brazil and the health-care secretariat of Belo Horizonte (state of Minas Gerais) (CAAE: 14038313.4.0000.5149). All participants signed the consent term.

### Data collection procedures

All data were collected by an examiner, a previously trained physical therapist, with the aid of another examiner, between May 2013 and May 2014 within 2 days (i.e. data from each patient was collected over a period of 2 days). Initial data were collected from the medical charts of the health-care unit; then, additional data were collected during a home visit. A previously elaborated assessment sheet was used to register the following:

- Variables related to health conditions and the assistance received at the health-care unit: date of stroke occurrence, associated diseases, names and number of drugs used, the services of the health-care unit used in the last 6 months before the assessment date, and whether or not the participant had received any information or guidance from a professional of the health-care unit about stroke management (source: medical charts and the participants or their caretakers).
- Variables related to personal factors such age, sex, educational background, socioeconomic status^17^, individual income, profession, the existence of a health insurance plan, present level of physical activity^18^ (source: participant or caretaker), and self-perception of health condition^19^ (source: participant).
- Variables related to environmental factors such as those surveyed in the Measure of the Quality of the Environment (MQE) questionnaire, in a version with 26 items translated and adapted for Brazilians with adequate measurement properties^20^. The MQE measured the participant’s perception about physical and social environments. It uses a seven-point scale, from -3 (important obstacle) to +3 (important facilitator)^20^^,^^21^. As recommended in the original paper, two final scores were calculated: the obstacle score (i.e. average of negative responses) and the environmental facilitator score (i.e. average of positive answers). The highest of the two scores (obstacle or facilitator) represented the participant’s perception environmental factors^20^^,^^21^.
- Variables of function and disability, organized according to the ICF, including body structure and function, activity, and participation^22^^-^^25^. For all of these variables, standard tests and measurements were used, with adequate measurement properties for stroke patients, which are commonly used in this population^22^^-^^48^. Table 1 shows all of the tests and measurements used, with the citations referring to their measurement properties that were adequate for stroke patient’s; the application procedures; and, the description of the interpretation/classification based on the scores, adopted according to the aforementioned previous recommendations^27^^-^^48^.

**Table 1:** Description of the tools used to assess functioning and disability variables, as well as of the measurement purposes and score interpretation.

**Tools**		**Measurement purpose and procedures applied**	**Score interpretation**	
**Body Structure and Function**				
GDS^27^^,^^28^^,^^29^		Depression Screening. 15-item questionnaire cross-cultural adapted to Portuguese-Brazil, applied under interview^27^^-^^29^		Total score obtained from summing the values of all items, and range from 0 to15. Total score >5 indicate positive depression screening^27^^,^^29^
FMS^30^^,^^31^		Motor impairment. Motor function was assessed by the examiner using the 36-items of the FMS^30^^,^^31^		Total score range from 0 to 100. Classification of the motor impairment: <50 severe, 50-84 marked, 85-95 moderate, 95-99 mild^30^^,^^31^
MAS^32^^,^^33^		Muscle Tone. Examiner assessed and rated the muscle tone following standardized procedures of the MAS. Muscles: elbow flexors and knee extensors^32^		Score range from 0 (No increase in muscle tone) to 4 (Affected part(s) rigid in flexion or extension)^32^^,^^33^
MST^34^^,^^45^		Muscle strength, assessed by the examiner within the modified sphygmomanometer. Muscles: hand grip, bilaterally. A single trial after familiarization, following standardized procedures^34^^,^^45^		Obtained valued was compared to the values of healthy subjects matched by age, gender and upper limb side (paretic/non-dominant and non-paretic/ dominant)^34^
MMS^36^^,^^37^		Cognitive function screening. Questionnaire cross-cultural adapted to Portuguese-Brazil, applied under interview^36^^,^^37^		Total score range from 0 to 30. Positive cognitive function screening: ≥13 illiterate; ≥18 1 to 7 years of schooling; ≥26 ≥8 years of schooling ^36^
**Activity**				
BBS^38^	Functional Balance. 14-item scale (14 functional activities to be performed) applied by the examiner^38^			Total score obtained from summing the values of all items, and range from 0 to 56. High risk of falling indicated by total scores ≤29^39^
ABILHAND^40^	Subject's perceived difficulty in performing everyday bimanual activities. 23-items questionnaire cross-cultural adapted to Portuguese-Brazil applied under interview^40^			The total score obtained from summing the values of all items was computed in a linear measure (logits) by the Rasch Analysis using a public software^40^^,^^41^
TUG^42^^,^^43^	Functional mobility. Examiner recorded the time used to stand up from a chair, walk 3 meters, turn around 180°, walk back to the chair and sit down. One trial after familiarization^42^^,^^43^			High risk of falling indicated by a test time ≥14s^44^
N-GS and M-GS^42^^,^^45^	Functional mobility. Examiner recorded the time used to walk the 5 meters in natural (N-GS) and maximal (M-GS) gait speed^42^^,^^45^			Complete community ambulation (N-GS>0.8 m/s), limited community ambulation (0,4<VM-N<0,8 m/s) and household ambulation (N-GS<0.4 m/s)**^46^**
**Participation**				
SSQOL-Brazil^47^	Specific QoL assessment. 49-items questionnaire cross-cultural adapted to Portuguese-Brazil, applied under interview^47^. QoL questionnaire most recommended to assess participation^48^			Total score obtained from summing the values of all items, and range from 49 to 245 (better perception of QoL)^47^

GDS: Geriatric Depression Scale; FMS: Fugl-Meyer Scale; MAS: Modified Ashworth Scale; MST: Modified Sphygmomanometer Test; MMS: Mini-Mental Scale; BBS: Berg Balance Scale; ABILHAND: Manual ability; NGS: Normal Gait Speed; MGS: Maximal Gait Speed; GS: Gait Speed; TUG: Timed Up and Go; Classif: classification; SSQOL: Stroke Specific Quality of Life Scale; QoL: Qualitiy of Life.

### Data analysis procedures

For the ordinal and nominal categorical variables, absolute and relative frequencies were calculated (%). For normally distributed quantitative variables (the Kolmogorov-Smirnov Test was used to verify data normality), medians and standard deviations (SDs) were calculated. For all other variables, the medians and interquartile differences were calculated (SPSS® for Windows, version 17.0; SPSS Inc., Chicago, IL, USA).

## Results

The sample was comprised of post-stroke patients who used the health-care unit identified by the family health-care teams and by the Family Health-Care Support Centre. Forty-four patients were assessed, with a predominance of women (54.5%, n=24). The patients average age was 69.23 years (SD, 13.12 years) and the time since the stroke was 67 months (SD, 66.52 months). Owing to the different limitations or disabilities presented by the participants, not all of them were able to undergo all tests and measurements (e.g., aphasia and potential alteration of cognitive function prevented the measurement of end points that involved the application of questionnaires based on self-reporting, such as self-perception of health, MQE, Geriatric Depression Scale, Manual Abilities [ABILHAND], and Stroke Specific Quality of Life [SSQOL] in eight [18.2%] participants). The number of participants assessed to obtain each end point is presented in the related tables (Tables 2-4).

**Table 2:** Descriptive data (mean (SD) or frequency (%)) of health condition variables of 44 stroke patients in one health care unit in Belo Horizonte, MG, Brazil.

**Variables**	**n=44**
Involvement side, % (n) Rigth Left Both sides	52.3 (23) 45.4 (20) 2.3 (1)
Episodes of stroke, % (n) One episode Two or more episodes	68.2 (30) 31.8 (14)
Stroke type, % (n) Ischemic Hemorrhagic No information on medical Record/Not able to inform	68.2 (30) 18.2 (8) 13.6 (6)
Associated Diseases, % (n)* Hypertension Hypercholesterolemia Visual Deficit Diabetes Others heart diseases (CI or AMI) Urinary Incontinence	81.8 (36) 56.8 (25) 52.3 (23) 27.3 (12) 27.3 (12) 25 (11)
Amount of medication, mean (SD)	4.11 (2.22)

* Have been reported associated diseases that showed higher frequency or equal to 25%.

CI: Cardiac Insufficiency; AMI: Acute Myocardial Infarction; SD: standard deviation; n: number.

**Table 4:** Descriptive data (mean (SD) or frequency (%)) of functioning and disability variables for stroke patients in one health care unit in Belo Horizonte, MG, Brazil.

**Variables**		**N**
Body Functions and Structures GDS: suspected depression % (n) FMS-Total: % (n) Mild impairment Moderate impairment Severe impairment Marked impairment Without impairment MAS: Elbow flexors/ Knee Extensors % (n) Score 0 Score 1 Score 1+ Score 2 Score 3 Score 4 HGS (*mmHg*), mean (SD) No paretic side-men/women Paretic side-men/women MMS: Negative test for changes in cognitive function % (n)	63 (22) 5 (2) 32 (13) 35 (14) 26 (10) 2 (1) 59/56.4 (23/22) 15.4/23.1 (6/9) 12.8/7.7 (5/3) 5.1/0 (2/0) 5.1/10.2 (2/4) 2.6/2.6 (1/1) 235.35/154.56 (63.11/48.79) 176/128.22 (75.04/50.42) 97.3 (36)	36 40 39 33 37
Activity BBS: With risk of falls % (n) ABILHAND (*logits*), mean (SD) NGS (*m/s*), mean (SD) MGS (*m/s*), mean (SD) GS-Classif.: Complete community ambulation Household ambulation Limited community ambulation TUG (*s*), mean (SD) TUG-Classif.: With risk of falls % (n)	51.4 (18) 2.39 (2.29) 0.77 (0.34) 1.08 (0.46) 41 (10) 12 (3) 47(11) 19.74 (10.9) 54 (13)	35 36 24* 24* 24* 24* 24*
Participation		
SSQOL, mean (SD)	164.21 (35.61)	36

GDS: Geriatric Depression Scale; FMS: Fugl-Meyer Scale; MAS: Modified Ashworth Scale; HGS: Hand Grip Strength; MMS: Mini-Mental Scale; BBS: Berg Balance Scale; ABILHAND: Manual ability; NGS: Normal Gait Speed; MGS: Maximal Gait Speed; GS: Gait Speed; TUG: Timed Up and Go; Classif: classification; SSQOL: Stroke Specific Quality of Life Scale; n: number; SD: standard deviation.

* Variables with the largest sample loss. Among the 44 subjects, 8 (18.2%) were bedridden and therefore physically unable to perform the tests, 3 (6.8%) were wheelchair dependents and therefore physically unable to perform the tests, 4 (9.1%) were not able to maintain the standing posture and again physically unable to perform the tests and 5 (11.4 %) refused to perform the tests (due to environmental limitations of their home they refused to move to another area to perform the tests).

### Health conditions and assistance received at the health-care unit

The features of the stroke episode (i.e. affected side, number of occurrences, and type of stroke), associated diseases, and number of drugs used are presented in Table 2.

By using the 6 months before the data collection as a reference, it was determined that the participants used the services of the health-care unit mainly to renew their drug prescriptions (54.5%, n=24). Other services used included appointments scheduled with a health-care professional (38.6%, n=17), routine checkups (28.3%, n=12), and vaccinations (20.5%, n=9).

Most participants, 72.7% (n=32), reported never having received information about stroke and the care associated with this condition at the health-care unit, nor during home appointments with professionals from the health-care unit nor from the Family Health-Care Support Centre. However, 79.5% (n=35) of participants stated that they believed it was important to receive information and clarifications in order to improve the health care they receive.

### Personal factors

The results related to personal factors are presented in Table 3. The most frequent results were as follows: female participants (54.5%), primary school education (43.2%), C1 socioeconomic status (40.9%) (the fifth category among eight categories of an economical classification from a Brazil association: *“Critérios de Classificação Econômica da Associação Brasileira de Empresas de Pesquisa-ABEP)*^17^, individual income of one minimum wage salary (70.5%), retired (88.6%), having no private health insurance (75%), having a sedentary lifestyle (86.4%), and having a reasonable health self-perception (47.2%).

**Table 3:** Descriptive data (mean (SD) or frequency (%)) of personal factors variables (n=44, except to self-perception of health n=36) in stroke patients in one health care unit in Belo Horizonte, MG, Brazil.

**Variables**	
Gender, female % (n)	54.5 (24)
Age *(years)*, mean (SD)	69.23 (13.12)
Education level, % (n)	
Complete Elementary School	43.2 (19)
Incomplete Elementary School	31.8 (14)
Can not read or write	11.4 (5)
Complete Middle School	6.8 (3)
Complete High School	4.5 (2)
Not studied, but can read and write	2.3 (1)
Socioeconomic Level, % (n)*	
Class C1	40.9 (18)
Class B2	22.7 (10)
Class C2	15.8 (7)
Class D	11.4 (5)
Class B1 and E (each)	4.6 (2)
Individual Income, % (n)	
One minimum wage	70.5 (31)
Between one and five minimum wages	20.5 (9)
Less than one minimum wage	9.1 (4)
Remunerated Activity, % (n)	
Retired	88.6 (39)
Unemployed	9.1 (4)
Remunerated Activity with fixed wage	2.3 (1)
Private Health Care, % (n) Not Physical Activity Level, % (n) Inactive Vigorous Insufficient Self-perception of Health, % (n) Reasonable Bad Very bad Great Very good Good	75 (33) 86.4 (38) 9.1 (4) 4.5 (2) 47.2 (17) 13.9 (5) 11.1 (4) 11.1 (4) 8.3 (3) 8.3 (3)

Class A1: 42-46 points; Class A2: 35-41 points; Class B1: 29-34 points; Class B2: 23-28 points; Class C1: 18-22 points; Class C2: 14-17 points; Class D: 8-13 points; Class E: 0-7 points.

* Classification according to *“Critérios de Classificação Econômica da Associação Brasileira de Empresas de Pesquisa”*^17^*.*

N: number; SD: standard deviation.

### Environmental factors

Considering the environment as a facilitator or obstacle, the average scores were 1.30 (SD, 0.41) points for facilitating factors and –2.40 (SD, 0.59) points for obstacles.

### Functioning and disability variables

The results for functioning and disability variables are provided in Table 4.

Concerning body structure and function variables, most participants tested negative for potential alteration of cognitive function (97.3%), positive for suspected depression (61.1%), severe to moderate motor impairment (67%), lack of changes in muscular tonus (>55%), and, on average, muscles weakness on the paretic side (men: 176 [SD, 75.04] mmHg and women: 128.88 [SD, 50.42] mmHg).

**Figure 1:**
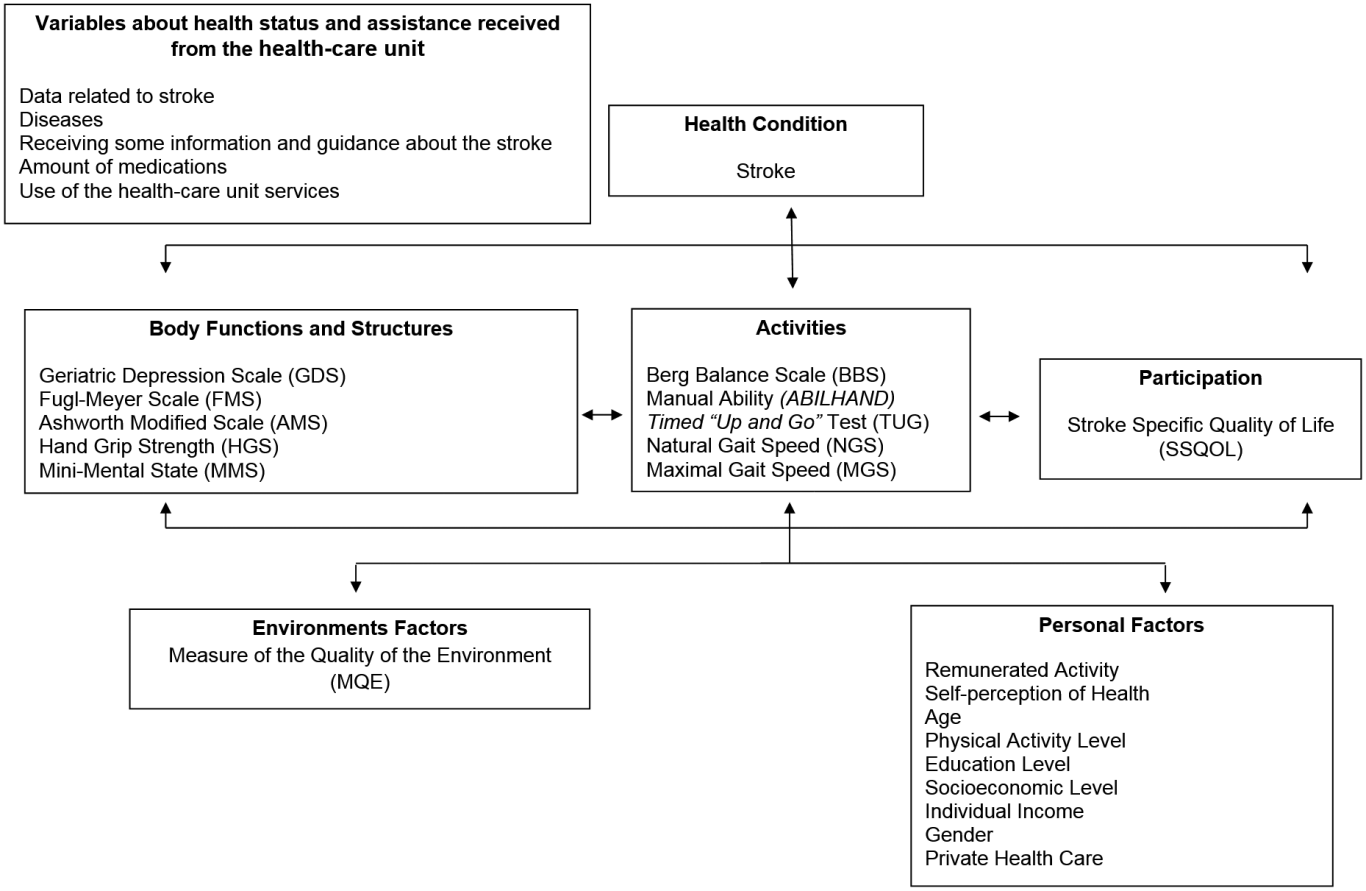
Variables and instruments used for data collection in stroke patients in one health care unit in Belo Horizonte, MG, Brazil, organized according to the conceptual framework of the International Classification of Functioning, Disability and Health (ICF).

Concerning activity and participation variables, most participants showed good self-perception of manual ability (2.39 [SD, 2.29] logits), were classified as having limited walking ability (88%), with the ability to improve natural gait speed, having a change in balance (51.43%), and functional mobility (54.16%), with indications of fall risks, and low perception of quality of life (average score of 164.21 [SD, 35.16] points in the SSQOL-Brazil). These results are summarized in Figure 1.

## Discussion

This study determined the function, disability, and health profile of stroke patients who were users of a primary health-care unit of the SUS located in the city of Belo Horizonte, Brazil. The participants showed chronic disabilities related to impairments in body structure and function, such as changes in motor function, limitations in certain activities, changes in mobility, increased risk of falling during functional activities, and restriction of participation. In addition, they perceived their environment to be an obstacle, which indicates requirement for multidisciplinary and continuous health care. For most participants, their health care was directed toward medical practices such as drug prescriptions, without access to practices related to health education and function.

The sociodemographic characteristics of the participants in this study, such as the predominance of women^9^^,^^14^, average age of approximately 70 years^9^^,^^12^^,^^14^, low educational level in most of the particpants^9^^,^^12^, and low individual income^9^^,^^12^^,^^14^, were similar to the results found in previous studies that investigated community participants who had suffered a stroke. The higher prevalence of women in the study (54.5%) might be related to the fact that women are more concerned about their health, and thus use health-care services more often, than men^49^^,^^50^. In addition, the average age of the assessed women fell within the post-menopausal years. During this period, women are at a higher risk of developing cardiovascular diseases, such as stroke, owing to a deficiency of estrogen and alterations in lipid metabolism^51^. Moreover, most of the participants also had a low educational level and economic status. It has been proposed that low educational level and low income could be factors that limit access to information on health conditions and the understanding of prescriptions, treatments, and care that chronic diseases, such as stroke, require. This could result in an insufficient control and follow-up of the disease and health care^50^^,^^52^. These features are highly relevant to health-care professionals, as they influence the attitude of patients toward self-care and treatment compliance.

Following a stroke, patients usually develop motor impairments^4^, which are associated with a decrease in function. Considering the scores obtained in the Fugl-Meyer Assessment^30^^,^^31^, most patients showed compromised motor abilities (classified as severe to moderate, 67%)^30^^,^^31^. In other studies in which the presence of post stroke impairments were assessed, chronic motor changes were also reported^4^.

One of the main impairments that occur after a stroke is hemiparesis. The assessed participants showed this alteration, characterized by a decrease in handgrip strength (HS) on the affected side. In stroke patients, the impairments in HS has been associated with worse performances in activities of daily living^53^ such as eating, getting dressed, or holding objects. Given the importance of HS as an indicator of health and function^35^^,^^53^, and the existing scientific evidence concerning the benefits of strengthening to improve the muscular force and function of stroke patients^54^, it is essential that these patients participate in rehabilitation programs that include progressive muscular strengthening. Such programs can be offered in the community and to groups, which increase the use of rehabilitation services and promote social interactions among patients, in addition to being viable strategies to promote regular physical activity. Finally, these are low-cost strategies when compared with other programs that require individual supervision^55^.

Despite the importance of physical activity in preventing a worsening of their health condition, decreasing the complications caused by the chronicity of stroke, improving patient functioning, controlling the risk factors associated with a new occurrence of stroke, and promoting health and functionality^55^^,^^56^, most of the participants were classified as inactive. The clinical practice guidelines related to the rehabilitation of stroke patients recommend participation in continuous physical exercise programs offered in the community^5^^,^^6^, which was not observed in this study.

For most patients, functional independence and community participation mean having the capacity for ambulation^46^. Often, stroke patients present sequelae that can alter their ambulation capacity. Thus, one of the main goals of rehabilitation is to increase or maintain the capacity to walk at home and in the community^43^, as walking speed is an important end point that reflects mobility^46^^,^^57^^,^^58^. In the test of the natural and maximum walking speed (WS-Natural and WS-Maximum, respectively), stroke patients have shown an average speed of 0.77 and 1.08 m/s, respectively. Similar results to those found for WS-Natural were reported in studies that associated WS with community ambulation in stroke patients^57^^,^^58^. As stroke patients tend to walk more slowly, it is necessary to determine if they are able to increase their speed when required in a specific community situation^57^, such as crossing the street, which is determined by assessing the WS-Maximum. The patients in this study showed this ability of increasing their walking speed since, in average, their natural walking speed was 0.77 m/s and their maximal walking speed was 1.08 m/s.

Another measure used to assess mobility was the timed “up and go” (TUG) test^42^^,^^43^. Considering the classification proposed for the TUG test^44^, most of the patients (54.16%) were classified as having a fall risk. In addition, when using the classification proposed for the Berg Balance Scale^39^, most patients (51.43%) were classified as having a fall risk. Among the adverse effects of falls are functional decline and an increased risk for new fall events^59^. In patients who already show chronic disabilities due to stroke, the effects of falls can further worsen their health condition, resulting in new hospitalizations and the need for extra care, which causes a burden to both patients and the public health-care system^59^.

SSQOL-Brazil is the most recommended life quality instrument for assessing the participation of stroke patients^48^. The assessed patients in the present study showed an average of 164 points, similar to those in previous studies associating this variable with a low perception of life quality^47^^,^^60^. In another study, in which a sample of stroke patients was compared to a paired control group, it was observed that the restriction in participation was higher among stroke subjects, and that they were 40% more likely to be restricted in their participation due to the effects of the stroke than people who had not suffered a stroke^61^.

Body structure and function, activity, and participation are variables that interact with each other and are associated with contextual factors, determining the functioning and/or disability of patients^7^. Considering the result obtained in the present study with the MQE^20^^,^^21^, the patients perceived the context around them as an obstacle. Rochette et al.^62^ also assessed the perception of stroke patients concerning environmental factors by using the MQE 6 months after the patients had been discharged from a rehabilitation unit in Canada. In their study, the patients mainly perceived the environment as a facilitator. Although the study assessed patients with the same health condition (stroke), the differences found between that study and the present study can be explained by the fact that the environment has different effects on people, depending on the severity of their health condition and what people did in the context in which they lived.

Most of the participants in this study (54.5%) used the services of the assigned health-care unit in the 6 months preceding the data collection, to renew their medical prescriptions. This result reflects the reality of the medicalization of care, in which the use of drugs is considered as the main therapeutic action by both physicians and patients. It also reflects the orientation of care to medical diagnosis only^63^^,^^64^. Educational actions can be used as a tool to inform patients about the possibility of non-medication treatments that can promote health and functioning and prevent the worsening of the current health condition. These treatment possibilities are available in the health-care system; however, they need to be articulated and organized in order to meet the specific demands of stroke patients. In this study, most participants reported never having received information or clarification on stroke and its appropriate care, although they considered such information important. Similar results were reported in a study related to educating patients with chronic diseases, in which users of primary health care considered guidance and education on health to be important factors in promoting self-care and treatment compliance^63^^,^^64^. Patients with chronic diseases require constant and continuous assistance that goes beyond the prescription of drugs and healing assistance. Therefore, as a component of health-care services for stroke patients, it is essential to plan and to develop educational practices that will increase the knowledge of patients and encourage them to be co-responsible in their own treatment^65^.

Although the health-care unit selected for this study complied with the criteria established by the MH, and had adequate resources and rehabilitation professionals, including a dedicated physical therapist from the Family Health-Care Support Centre, the results showed that the participants still had relevant disabilities and health needs that were not met. This poses the question of why the clinical practice recommendations for the rehabilitation of stroke patients ^5^^,^^6^ have still not been effectively implemented. It also raised the question of why the scientific evidence that directs the implementation of continuous physical exercise programs, which could be easily offered in the community^5^^,^^6^^,^^54^^-^^56^, are not being used to guide professional practice. This context highlights the importance of involving indicators, measurement instruments, and strategies guided by the Model of Functioning and Disability model related to the ICF^7^ in the actions of professionals of the family health-care teams and of the Family Health Support Centre^66^^,^^67^, so that the assistance provided to patients is directed to essential questions that have not yet been considered.

The results of this study clearly showed the potential importance of the role of health-care professionals in the functional recovery and systematic follow-up of stroke patients. However, it is extremely important that the primary actions are directed to preventing the risk factors for the development of stroke and other disabling chronic diseases, and to promoting health targeted toward the entire population. The need for these actions are clearly highlighted in the basics of primary care, family health-care teams, and the Family Health-Care Support Centre, in addition to the list of goals of the “Guidelines for the Care of People with Chronic Disease in Health-Care Networks and Priority Care Lines” of the MH, “contributing to the promotion of health in the population and preventing the development of chronic diseases and their complications”^68^^:11^.

A relevant limitation of this study was the inclusion of participants from only one health-care unit. However, as this is the first study to describe the functional profile of patients considering the biopsychosocial model of a nonconvenience sample, the results add important information that can guide future studies and actions related to the function and health of stroke patients.

## Conclusion

Stroke patients who use the SUS primary health-care services, specifically one health-care unit in the city of Belo Horizonte, Brazil showed chronic disabilities related to impairments in body structure and function, limitations in activities, and restriction in participation. Their health care was directed toward medication treatment rather than practices related to health education, assistance in recovering functionality, and promotion of health and functionality. The health needs presented by these patients should be taken into consideration by health-care professionals, in order to organize the continuous health care process for stroke patients, and by researchers, in order to develop future evidence based studies. The results of this study are expected to enable the discussion, planning, and implementation of strategies for health care that involve follow-up and care directed specifically to the stroke patient population, on the basis of current evidence that complies with the recommendations in the guidelines of clinical practices and care of the MH. In this study, such strategies were not observed, despite including a sample from a population that attended a health-care unit with the potential for providing such assistance. Furthermore, the results of this study are also expected to reinforce the importance of disease-preventing and health-promoting actions directed toward all individuals, in order to decrease the occurrence of chronic and debilitating diseases such as stroke.

## References

[1] 1 Feigin VL, Lawes CM, Bennett DA, Barker-Collo SL, Parag V. Worldwide stroke incidence and early case fatality reported in 56 population-based studies: a systematic review. Lancet Neurol. 2009;8(4):355-69. http://dx.doi.org/10.1016/S1474-4422(09)70025-0. 10.1016/S1474-4422(09)70025-0 19233729

[2] 2 Mozaffarian D, Benjamin EJ, Go AS, Arnett DK, Blaha MJ, Cushman M, et al. Heart disease and stroke statistics-2016 update: a report from the American Heart Association. Circulation. 2016;133(4):e38-360. http://dx.doi.org/10.1161/CIR.0000000000000366. 10.1161/CIR.0000000000000350 26673558

[3] 3 Young J, Forster A. Review of stroke rehabilitation. BMJ. 2007;334:86-90. http://dx.doi.org/10.1136/bmj.39059.456794.68. 10.1136/bmj.39059.456794.68 PMC1767284 17218714

[4] 4 Arene N, Hidler J. Understanding motor impairment in the paretic lower limb after a stroke: a review of the literature. Top Stroke Rehabil. 2009;16(5):346-56. http://dx.doi.org/10.1310/tsr1605-346. 10.1310/tsr1605-346 19903653

[5] 5 Ottawa P, Khadilkar A, Phillips K, Jean N, Lamothe C, Milne S, et al. Ottawa panel evidence-based clinical practice guidelines for post-stroke rehabilitation. Top Stroke Rehabil. 2006;13(2):1-269. http://dx.doi.org/10.1310/3TKX-7XEC-2DTG-XQKH. 10.1310/3TKX-7XEC-2DTG-XQKH 16939981

[6] 6 National Stroke Foundation. Clinical guidelines for stroke management. Melbourne: National Stroke Foundation; 2010.

[7] 7 World Health Organization – WHO. International Classification of Functioning, Disability and Health – ICF. Geneva: WHO; 2001.

[8] 8 Lexell J, Brogårdh C. The use of ICF in the neurorehabilitation process. NeuroRehabilitation. 2015;36(1):5-9. 10.3233/NRE-141184 25547759

[9] 9 Leite HR, Nunes APN, Corrêa CL. Epidemiological profile of stroke survivors registered at the health family strategy of Diamantina, MG. Arq Cienc Saúde. 2011;15(1):15-21.

[10] 10 Mazzola D, Polese JC, Schuster RC, Oliveira SG. Perfil dos pacientes acometidos por acidente vascular encefálico assistidos na clínica de fisioterapia neurológica da universidade de passo fundo. RBPS. 2007;20(1):22-7. http://dx.doi.org/10.5020/18061230.2007.p22.

[11] 11 Polese JC, Tonial A, Jung FK, Mazuco R, Oliveira SG, Schuster RC. Avaliação da funcionalidade de indivíduos acometidos por Acidente Vascular Encefálico. Rev Neurocienc. 2008;16(3):175-8.

[12] 12 Lopes Junior JEG, Freitas Júnior JHA, Figueiredo ADJ, Santana FM. Perfil dos Pacientes Acometidos por Acidente Vascular Encefálico Cadastrados na Estratégia de Saúde da Família. Rev Fisioter S Fun Fortaleza. 2013;2(1):21-7.

[13] 13 Leite HR, Nunes APN, Correa CL. Perfil epidemiológico de pacientes acometidos por acidente vascular encefálico cadastrados na estratégia de saúde da família em Diamantina, MG. Fisioter Pesqui. 2009;16(1):34-9.

[14] 14 Ribeiro KSQ, Neves RF, Brito GEG, Morais JD, Lucena EMF, Medeiros JM, et al. Profile of users affected by stroke followed by the Family Health Strategy in a capital of northeastern Brazil. Rev Bras Ci Saúde. 2012;16(s2):25-44.

[15] 15 Brasil. Ministério da Saúde. Departamento de Atenção Básica. Portal da Saúde. Equipe de Saúde da Família [Internet]. Brasília: MS; 2015 [cited 2015 Nov 17]. Available from: http://dab.saude.gov.br/portaldab/smp_como_funciona.php?conteudo=esf

[16] 16 Figueiredo EN. Estratégia saúde da família e núcleo de apoio à saúde da família: diretrizes e fundamentos. São Paulo: UNA-SUS; 2010. 21 p.

[17] 17 Associação Brasileira de Empresas de Pesquisas – ABEP. Dados com base no levantamento sócio econômico [Internet]. São Paulo: ABEP; 2014 [cited 2014 Out 14]. Available from: www.apeb.org

[18] 18 Centers for Disease Control and Prevention. Physical activity trends: United States, 1990–1998. MMWR Morb Mortal Wkly Rep. 2001;50(9):166-9. 11393487

[19] 19 Pavao AL, Werneck GL, Campos MR. Self-rated health and the association with social and demographic factors, health behavior, and morbidity: a national health survey. Cad Saude Publica. 2013;29(4):723-34. 23568302

[20] 20 Basílio ML, Teixeira-Salmela LF. Cross-cultural adaptation and reproducibility of the measure of the quality of the environment in individuals with stroke. In: Anais do X Congresso Brasileiro de Doenças Cerebrovasculares – AVC2015; 2015, Belo Horizonte, Brazil. São Paulo: Academia Brasileira de Neurologia; 2015. (Arquivos de Neuropsiquiatria. 73(S1):61-61).

[21] 21 Fougeyrollas P, Noureau L, St-Michael G, Boschen K. Measure of the quality of the environment: version 2.0. Québec: RIPPH/INDCP; 2008.

[22] 22 Salter K, Jutai JW, Teasell R, Foley NC, Bitensky J. Issues for selection of outcome measures in stroke rehabilitation: ICF Body Functions. Disabil Rehabil. 2005;27(4):191-207. http://dx.doi.org/10.1080/09638280400008537. 10.1080/09638280400008537 15824050

[23] 23 Salter K, Jutai JW, Teasell R, Foley NC, Bitensky J, Bayley M. Issues for selection of outcome measures in stroke rehabilitation: ICF Activity. Disabil Rehabil. 2005;27(6):315-40. http://dx.doi.org/10.1080/09638280400008545. 10.1080/09638280400008545 16040533

[24] 24 Salter K, Jutai JW, Teasell R, Foley NC, Bitensky J, Bayley M. Issues for selection of outcome measures in stroke rehabilitation: ICF Participation. Disabil Rehabil. 2005;27(9):507-28. http://dx.doi.org/10.1080/0963828040008552. 10.1080/0963828040008552 16040555

[25] 25 Schepers VP, Ketelaar M, van de Port IG, Visser-Meily JM, Lindeman E. Comparing contents of functional outcome measures in stroke rehabilitation using the International Classification of Functioning, Disability and Health. Disabil Rehabil. 2007;29(3):221-30. http://dx.doi.org/10.1080/09638280600756257. 10.1080/09638280600756257 17364773

[26] 26 Duncan PW. Outcome measures in stroke rehabilitation. Handb Clin Neurol. 2013;110:105-11. http://dx.doi.org/10.1016/B978-0-444-52901-5.00009-5. 10.1016/B978-0-444-52901-5.00009-5 23312634

[27] 27 Almeida OP, Almeida SA. Reliability of the Brazilian version of the ++abbreviated form of Geriatric Depression Scale (GDS) short form. Arq Neuropsiquiatr. 1999;57(2B):421-6. http://dx.doi.org/10.1590/S0004-282X1999000300013. 10.1590/s0004-282x1999000300013 10450349

[28] 28 Burton LJ, Tyson S. Screening for mood disorders after stroke: a systematic review of psychometric properties and clinical utility. Psychol Med. 2015;45(1):29-49. http://dx.doi.org/10.1017/S0033291714000336. 10.1017/S0033291714000336 25066635

[29] 29 Castelo MS, Coelho-Filho JM, Carvalho AF, Lima JW, Noleto JC, Ribeiro KG, et al. Validity of the Brazilian version of the Geriatric Depression Scale (GDS) among primary care patients. Int Psychogeriatr. 2010;22(1):109-13. http://dx.doi.org/10.1017/S1041610209991219. 10.1017/S1041610209991219 19883523

[30] 30 Maki T, Quagliato EMAB, Cacho EWA, Paz LPS, Nascimento NH, Inoue MMEA, et al. Reliability Study on the Application of the Fugl-Meyer Scale in Brazil. Rev Bras Fisioter. 2006;10(2):177-83. http://dx.doi.org/10.1590/S1413-35552006000200007.

[31] 31 Michaelsen S, Rocha A, Knabben R, Rodrigues L, Fernandes C. Translation, adaptation and inter-rater reliability of the administration manual for the Fugl-Meyer scale. Rev Bras Fisioter. 2011;15(1):80-8. http://dx.doi.org/10.1590/S1413-35552011000100013. 21519719

[32] 32 Bohannon RW, Smith MB. Interrater reliability of a modified Ashworth scale of muscle spasticity. Phys Ther. 1987;67(2):206-7. 10.1093/ptj/67.2.206 3809245

[33] 33 Li F, Wu Y, Li X. Test-retest reliability and inter-rater reliability of the Modified Tardieu Scale and the Modified Ashworth Scale in hemiplegic patients with stroke. Eur J Phys Rehabil Med. 2014;50(1):9-15. 24309501

[34] 34 Martins JC, Teixeira-Salmela LF, Castro e Souza LA, Aguiar LT, Lara EM, Moura JB, et al. Reliability and validity of the modified sphygmomanometer test for the assessment of strength of upper limb muscles after stroke. J Rehabil Med. 2015;47(8):697-705. http://dx.doi.org/10.2340/16501977-1978. 10.2340/16501977-1978 26035840

[35] 35 Martins JC, Aguiar LT, Lara EM, Teixeira-Salmela LF, Faria CDCM. Assessment of grip strength with the modified sphygmomanometer test: association between upper limb global strength and motor function. Braz J Phys Ther. 2015;19(6):498-506. http://dx.doi.org/10.1590/bjpt-rbf.2014.0118. 10.1590/bjpt-rbf.2014.0118 PMC4668344 26647752

[36] 36 Bertolucci PH, Brucki SM, Campacci SR, Juliano Y. The Mini-Mental State Examination in a general population: impact of educational status. Arq Neuropsiquiatr. 1994;52(1):1-7. http://dx.doi.org/10.1590/S0004-282X1994000100001. 8002795

[37] 37 Cumming TB, Churilov L, Linden T, Bernhardt J. Montreal cognitive assessment and Mini-Mental State Examination are both valid cognitive tools in stroke. Acta Neurol Scand. 2013;128(2):122-9. http://dx.doi.org/10.1111/ane.12084. 10.1111/ane.12084 23425001

[38] 38 Miyamoto ST, Lombardi JI, Berg KO, Ramos LR, Natour J. Brazilian version of the Berg balance scale. Braz J Med Biol Res. 2004;37(9):1411-21. http://dx.doi.org/10.1590/S0100-879X2004000900017. 10.1590/s0100-879x2004000900017 15334208

[39] 39 Maeda N, Kato J, Shimada T. Predicting the probability for fall incidence in stroke patients using the Berg Balance Scale. J Int Med Res. 2009;37(3):697-704. http://dx.doi.org/10.1177/147323000903700313. 10.1177/147323000903700313 19589253

[40] 40 Basílio ML, Faria-Fortini I, Magalhães LC, Assumpção FSN, Carvalho AC, Teixeira-Salmela LF. Cross-cultural validity of the Brazilian version of the ABILHAND questionnaire for chronic stroke individuals, based on Rasch analysis. J Rehabil Med. 2015;48(1):6-13. http://dx.doi.org/10.2340/16501977-2044. 10.2340/16501977-2044 26660946

[41] 41 Rehab-scales.org. ABILHAND: a measure of manual ability for adults with upper limb impairment [Internet]. Montignies-sur-Sambre: Rehab-scales.org; 2014 [cited 2014 Nov 7]. Available from: http://www.rehab-scales.org/abilhand.html

[42] 42 Faria CD, Teixeira-Salmela LF, Gomes M No, Rodrigues-de-Paula F. Performance-based tests in subjects with stroke: outcome scores, reliability and measurement errors. Clin Rehabil. 2012;26(5):460-9. http://dx.doi.org/10.1177/0269215511423849. 10.1177/0269215511423849 22008883

[43] 43 Hafsteinsdottir TB, Rensink M, Schuurmans M. Clinimetric properties of the Timed Up and Go Test for patients with stroke: a systematic review. Top Stroke Rehabil. 2014;21(3):197-210. http://dx.doi.org/10.1310/tsr2103-197. 10.1310/tsr2103-197 24985387

[44] 44 Andersson AG, Kamwendo K, Seiger A, Appelros P. How to identify potential fallers in a stroke unit: validity indexes of 4 test methods. J Rehabil Med. 2006;38(3):186-91. http://dx.doi.org/10.1080/16501970500478023. 10.1080/16501970500478023 16702086

[45] 45 Nascimento LR, Caetano LC, Freitas DC, Morais TM, Polese JC, Teixeira-Salmela LF. Different instructions during the ten-meter walking test determined significant increases in maximum gait speed in individuals with chronic hemiparesis. Rev Bras Fisioter. 2012;16(2):122-7. http://dx.doi.org/10.1590/S1413-35552012005000008. 10.1590/s1413-35552012005000008 22378478

[46] 46 Perry J, Garrett M, Gronley JK, Mulroy S. Classification of Walking Handicap in the Stroke Population. Stroke. 1995;26(6):982-9. http://dx.doi.org/10.1161/01.STR.26.6.982. 10.1161/01.str.26.6.982 7762050

[47] 47 Lima R, Teixeira-Salmela LF, Magalhães L, Gomes M No. Psychometric properties of the Brazilian version of the Stroke Specifi c Quality of Life Scale: application of the Rasch model. Rev Bras Fisioter. 2008;12(2):149-56. http://dx.doi.org/10.1590/S1413-35552008000200012.

[48] 48 Faria CD, Silva SM, Correa JC, Laurentino GE, Teixeira-Salmela LF. Identification of ICF participation categories in quality-of-life instruments utilized in cerebrovascular accident victims. Rev Panam Salud Publica. 2012;31(4):338-44. 22652975

[49] 49 Alves RF, Silva RP, Ernesto MV, Lima AGB, Souza FM. Gênero e saúde: o cuidar do homem em debate. Psicol Teor Prat. 2011;13(3):152-66.

[50] 50 Taveira LF, Pierin AMG. O nível socioeconômico pode influenciar as características de um grupo de hipertensos? Rev Latino-am Enfermagem. 2007;15(5). 10.1590/s0104-11692007000500008 18157444

[51] 51 Magalhães CK. Alterações cardiovasculares na menopausa: dificuldades no manejo dos fatores de risco. Revista da SOCERJ. 2001;14(4):321-6.

[52] 52 Rodrigues FFL, Santos MA, Teixeira CRS, Gonela JT, Zanetti ML. Relação entre conhecimento, atitude, escolaridade e tempo de doença em indivíduos com diabetes mellitus. Acta Paul Enferm. 2012;25(2):284-90. http://dx.doi.org/10.1590/S0103-21002012000200020.

[53] 53 Harris JE, Eng JJ. Paretic upper-limb strength best explains arm activity in people with stroke. Phys Ther. 2007;87(1):88-97. http://dx.doi.org/10.2522/ptj.20060065. 10.2522/ptj.20060065 17179441

[54] 54 Harris JE, Eng JJ. Strength training improves upper-limb function in individuals with stroke: a meta-analysis. Stroke. 2010;41(1):136-40. http://dx.doi.org/10.1161/STROKEAHA.109.567438. 10.1161/STROKEAHA.109.567438 19940277

[55] 55 Billinger SA, Arena R, Bernhardt J, Eng JJ, Franklin BA, Johnson CM, et al. Physical activity and exercise recommendations for stroke survivors: a statement for healthcare professionals from the American Heart Association/American Stroke Association. Stroke. 2014;45(8):2532-53. http://dx.doi.org/10.1161/STR.0000000000000022. 10.1161/STR.0000000000000022 24846875

[56] 56 Saunders DH, Greig CA, Mead GE. Physical activity and exercise after stroke: review of multiple meaningful benefits. Stroke. 2014;45(12):3742-7. http://dx.doi.org/10.1161/STROKEAHA.114.004311. 10.1161/STROKEAHA.114.004311 25370588

[57] 57 van de Port IG, Kwakkel G, Lindeman E. Community ambulation in patients with chronic stroke: how is it related to gait speed? J Rehabil Med. 2008;40(1):23-7. http://dx.doi.org/10.2340/16501977-0114. 10.2340/16501977-0114 18176733

[58] 58 Taylor-Piliae RE, Latt LD, Hepworth JT, Coull BM. Predictors of gait velocity among community-dwelling stroke survivors. Gait Posture. 2012;35(3):395-9. http://dx.doi.org/10.1016/j.gaitpost.2011.10.358. 10.1016/j.gaitpost.2011.10.358 PMC4696768 22119886

[59] 59 Maia B, Viana P, Arantes P, Alencar M. Consequências das quedas em idosos vivendo na comunidade: revisão sistemática. Rev Bras Geriatr Gerontol. 2011;14(2):381-94. http://dx.doi.org/10.1590/S1809-98232011000200017.

[60] 60 Monteiro RB, Laurentino GE, Melo PG, Cabral DL, Correa JC, Teixeira-Salmela LF. Fear of falling and the relationship with the measure of functional independence and quality of life in post-Cerebral Vascular Accident (Stroke) victims. Cien Saude Colet. 2013;18(7):2017-27. http://dx.doi.org/10.1590/S1413-81232013000700017. 10.1590/s1413-81232013000700017 23827906

[61] 61 Skolarus LE, Burke JF, Brown DL, Freedman VA. Understanding stroke survivorship: expanding the concept of poststroke disability. Stroke. 2014;45(1):224-30. http://dx.doi.org/10.1161/STROKEAHA.113.002874. 10.1161/STROKEAHA.113.002874 PMC3939034 24281223

[62] 62 Rochette A, Desrosiers J, Noreau L. Association between personal and environmental factors and the occurrence of handicap situations following a stroke. Disabil Rehabil. 2001;23(13):559-69. http://dx.doi.org/10.1080/09638280010022540. 10.1080/09638280010022540 11451190

[63] 63 Bezerra IC, Jorge MSB, Gondim APS, Lima LL, Vasconcelos MGF. “I went to the health unit and the doctor sent me here”: process of medicationalization and (non)resolution of mental healthcare within primary care. Interface. 2014;18(48):61-74. http://dx.doi.org/10.1590/1807-57622013.0650.

[64] 64 Tesser CD, Poli NP, Campos GW. User embracement and social (de)medicalization: a challenge for the family health teams. Cien Saude Colet. 2010;15(Suppl 3):3615-24. http://dx.doi.org/10.1590/S1413-81232010000900036. 10.1590/s1413-81232010000900036 21120349

[65] 65 Taddeo P, Gomes K, Caprara A, Gomes A, Oliveira G, Moreira T. Acesso, prática educativa e empoderamento de pacientes com doenças crônicas. Ciênc. Saúde Coletiva. 2012;17(11):2923-30. http://dx.doi.org/10.1590/S1413-81232012001100009. 10.1590/s1413-81232012001100009 23175299

[66] 66 Tempest S, McIntyre A. Using the ICF to clarify team roles and demonstrate clinical reasoning in strokerehabilitation. Disabil Rehabil. 2006;28(10):663-7. http://dx.doi.org/10.1080/09638280500276992. 10.1080/09638280500276992 16690581

[67] 67 Neubert S, Sabariego C, Stier-Jarmer M, Cieza A. Development of an ICF-based patient education program. Patient Educ Couns. 2011;84(2):e13-7. http://dx.doi.org/10.1016/j.pec.2010.07.021. 10.1016/j.pec.2010.07.021 20705411

[68] 68 Brasil. Ministério da Saúde. Secretaria de Atenção à Saúde. Departamento de Atenção Básica. Diretrizes para o cuidado das pessoas com doenças crônicas nas redes de atenção à saúde e nas linhas de cuidado prioritárias. Brasília: Ministério da Saúde; 2013. v. 28, il.

